# Supplementary material for: Dynalign II: common secondary structure prediction for RNA homologs with domain insertions
Source: Nucleic Acids Res. 2014 Nov 21;42(22):13939–48. doi: 10.1093/nar/gku1172 (PMC4267632; doi:10.1093/nar/gku1172)
Supplement: SUPPLEMENTARY DATA [file supp_gku1172_nar-02021-z-2014-File012.zip › manual/javadoc/comments/Overview.html]

These are the Javadocs for all Java folders in the RNAstructure software package.
  
These folders encompass the Java drawing framework, the Java RNAstructure interface, and the Java utilities classes.
  
  
Some classes and packages are backed by C++ files; these C++ files are documented with Doxygen documentation.
